# CRISPR-Cas9 *In Situ* engineering of subtilisin E in *Bacillus subtilis*

**DOI:** 10.1371/journal.pone.0210121

**Published:** 2019-01-07

**Authors:** Marcus A. Price, Rita Cruz, Scott Baxter, Franck Escalettes, Susan J. Rosser

**Affiliations:** 1 Department of Quantitative Biology, Biochemistry and Biotechnology, University of Edinburgh, Edinburgh, United Kingdom; 2 Ingenza Ltd., Roslin Innovation Centre, Roslin, United Kingdom; University of Florida, UNITED STATES

## Abstract

CRISPR-Cas systems have become widely used across all fields of biology as a genome engineering tool. With its recent demonstration in the Gram positive industrial workhorse *Bacillus subtilis*, this tool has become an attractive option for rapid, markerless strain engineering of industrial production hosts. Previously described strategies for CRISPR-Cas9 genome editing in *B*. *subtilis* have involved chromosomal integrations of Cas9 and single guide RNA expression cassettes, or construction of large plasmids for simultaneous transformation of both single guide RNA and donor DNA. Here we use a flexible, co-transformation approach where the single guide RNA is inserted in a plasmid for Cas9 co-expression, and the donor DNA is supplied as a linear PCR product observing an editing efficiency of 76%. This allowed multiple, rapid rounds of *in situ* editing of the subtilisin E gene to incorporate a salt bridge triad present in the *Bacillus clausii* thermotolerant homolog, M-protease. A novel subtilisin E variant was obtained with increased thermotolerance and activity.

## Introduction

The clustered regularly interspaced short palindromic repeats (CRISPR)-associated (Cas) systems of adaptive immunity in bacteria have become widely used across all fields of biology as a genome editing tool since demonstration of its use as a RNA-guided DNA endonuclease in 2012 [1]. The technique, based on the type II CRISPR-Cas9 system from *Streptococcus pyogenes*, makes use of the host DNA double-strand break (DSB) repair machinery to introduce mutations within the DNA sequence. DSBs can be repaired by the error-prone non-homologous end joining (NHEJ) or homology directed DNA repair (HDR) mechanisms of the cell. NHEJ can introduce random mutations during repair, while HDR allows the introduction of selected modifications in the presence of an engineered template with chromosome homology regions flanking the desired mutations. Uptake of CRISPR-Cas9 genome editing in the Gram positive model organism *Bacillus subtilis* was however relatively slow, perhaps, in part, due to the high number of well-established genomic modification tools already available. However, recently several publications were released in quick succession showing the development of the CRISPR-Cas9 system in this host [2–6]. These initially showed use of the catalytically active Cas9 for large scale genomic deletions, small and large DNA insertions, gene silencing by introduction of a stop codon and engineering of increased resistance to bacteriophage SPP1 [2–6]. Additionally, the catalytically inactivated ‘dead’ Cas9 (dCas9) was used to allow phenotypic analysis of all essential genes in *B*. *subtilis*. This was performed by dCas9 binding at the target region within the gene and not inducing a DSB, subsequently reducing the level of transcription from that gene and any other genes downstream in the same operon. [7].

Two methodologies have been exemplified in *B*. *subtilis* using the catalytically active Cas9. First, Westbrook *et al*. (2016) demonstrated a system where a single plasmid simultaneously delivers an integration cassette for expression of the chromosome targeting crRNA and the donor DNA (dDNA) for DSB repair, in a strain where the *cas9* gene and tracrRNA, which associates Cas9 to the crRNA, was previously integrated in the chromosome [3]. Others have used an alternative system where *cas9* and sgRNA (single guide RNA where tracrRNA and crRNA are combined as a single fragment) expression cassettes, as well as dDNA, are delivered in a single temperature sensitive plasmid [2,5,6]. With the first method, the crRNA expression cassette must be removed with a subsequent transformation step to restore the integration site before next rounds of editing are possible. The second strategy, although more amenable to multiple rounds of editing, involves a 2-step construction of a large shuttle vector to introduce the sgRNA and dDNA [2]. Here, we present a simple and straightforward method which allows rapid *in situ* edits of the genome, using a plasmid which can target any loci within *B*. *subtilis* in a single cloning step, and without previous genome modifications. During the editing process, this plasmid transcribes the sgRNA under the control of the strong constitutive promoter, P_veg_, and utilises the basal expression level of the P_grac_ promoter to regulate *cas9* expression. The dDNA in the form of a linear PCR product is co-transformed with the plasmid, providing the template for DSB repair.

The use of CRISPR-Cas9 mediated genome editing using this approach was first exemplified by knock-out of the α-amylase encoding gene, *amyE*. Subsequently our system was shown to be compatible for engineering of industrial production hosts by *in situ* modification of the *aprE* gene. This encodes for the industrially relevant enzyme subtilisin E, used globally within the detergent industry. As the wild type enzyme can be broken down by detergent formulations and heat, subtilisin variants with improved thermostability and pH tolerance have long been of interest. Subtilisin E has been widely used as a target for protein engineering experiments [8]. A salt-bridge triad (Arg19-Glu271-Arg275), identified in the subtilisin E homolog from *Bacillus clausii* (M-protease), was found to be a contributor for the characteristic thermotolerance of this enzyme [9]. Here, we use CRISPR-Cas9 mediated genome editing to replace the respective residues in subtilisin E (Gln125-Gln377-Gln381), and evaluate the effect of the salt bridge on the thermostability and activity of the new variant. This work illustrates the use of a simple CRISPR-Cas9 system for *B*. *subtilis* rapid, *in situ* protein engineering, which is at the core of industrial biotechnology to provide new, suitable and competitive biocatalysts.

## Materials and methods

### Strains and media

The strains and plasmids used in this study are listed in Table 1. All oligonucleotides used in this study are listed in S1 Table. *Escherichia coli* Top10 strains were used to construct recombinant plasmids. Bacterial cells were cultured in Lysogeny broth (LB) or LB agar (VWR) media at 37°C. Unless otherwise stated, the following antibiotics were added to the media when required: ampicillin (200 μg/mL), chloramphenicol (10 μg/mL), or spectinomycin (100 μg/mL).

**Table 1:** Strains and plasmids used in this study.

Strain/ Plasmid	Genotype	Reference
**Strains**		
***B*. *subtilis***		
168	*trpC2*	Laboratory stock
BAC0094	*B*. *subtilis* 168 with *aprE* Q125R variant.	This work
BAC0095	*B*. *subtilis* 168 with *aprE* Q377E and Q381R variant.	This work
BAC0097	BAC0095 with *aprE* Q125R, Q377E and Q381R variant	This work
BAC0114	*B*. *subtilis* 168 *ΔaprE*::*aad9*	This work
BAC0119	BAC0114, pBAC0059.	This work
BAC0120	BAC0114, pBAC0060.	This work
BAC0121	BAC0114, pBAC0068.	This work
BAC0122	BAC0114, pBAC0069.	This work
***E*. *coli***		
Top10	*F- mcrA Δ(mrr-hsdRMS-mcrBC) φ80lacZΔM15 ΔlacX74 recA1 araD139 Δ(araleu) 7697 galU galK rpsL* (Str^R^) *endA1 nupG*	Invitrogen
**Plasmids**		
pHT01	*E*. *coli/B*. *subtilis* shuttle vector carrying P_grac_ and *lacI*, *bla*, *cat*.	MoBiTec
pdCas9-bacteria	*tetR*; *dCas9* (*S*. *pyogenes*); rrnB T1; p15a ori; *cat*	[10]
pING0001	P_bad_; rrnB T1; rrnB T2; *bla;* pBR327 ori; *araC*	Ingenza Ltd.
pING0002	P_bad_; *aphAI;* pMB1 ori; *araC*	Ingenza Ltd.
pDR111	*bla*; 5’ *amyE*; *aad9*; P_spac_;*lacI*; 3’ *amyE*	[11]
pBAC0001	pHT01 with SapI sites removed.	This work
pBAC0008	pBAC0001 with rrnB T1 T2; sgRNA Cas9 handle; protospacer cloning site; P_veg_	This work
pBAC0013	pdCas9-bacteria with catalytically active *cas9*.	This work
pBAC0015	pBAC0008 with *cas9* (*S*. *pyogenes*) from pBAC0013. Cas9 expression regulated by the P_grac_ promoter.	This work
pBAC0027	pBAC0015 with amyE-1 sgRNA DNA.	This work
pBAC0035	pBAC0015 with non-targeting sgRNA DNA.	This work
pBAC0041	pBAC0015 with amyE-2 sgRNA DNA.	This work
pBAC0047	pBAC0015 with amyE-3 sgRNA DNA.	This work
pBAC0054	pBAC0015 with aprE-1 sgRNA DNA.	This work
pBAC0055	pBAC0015 with aprE-2 sgRNA DNA.	This work
pBAC0059	pHT01 with *aprE* (native) from *B*. *subtilis* 168 with ATG start codon.	This work
pBAC0060	pHT01 with *aprE* (Q125R, Q377E and Q381R variant) from BAC0097 with ATG start codon.	This work
pBAC0068	pHT01 with *aprE* (Q125R variant) from BAC0094 with ATG start codon.	This work
pBAC0069	pHT01 with *aprE* (Q377E and Q381R variant) from BAC0095 with ATG start codon.	This work

### Plasmid construction

Unless otherwise stated, plasmid construction was performed as described in Sambrook and Russell [12]. DNA oligonucleotides were purchased from Sigma-Aldrich. The reagents for PCR, restriction digest, Gibson assembly, DNA phosphorylation and ligation were purchased from New England Biolabs (NEB). DNA purification was performed using the kits from NEB. DNA sequences were confirmed by Source Bioscience.

pBAC0001 was constructed by Gibson assembly to remove the SapI sites present in pHT01 (MoBiTec) [13]. PCR products for Gibson assembly were prepared from pHT01 using oligonucleotides oMAP0002/0003/0004/0005.

pBAC0008 plasmid was constructed using the inABLE plasmid assembly method [14]. Individual 5' truncated parts were prepared by PCR followed by restriction digest at 5’ and 3’ regions with SapI. These parts were ligated to phosphorylated and annealed oligonucleotides at each terminus, containing 3 nt and 16 nt single strands at the 5' and 3' ends, respectively. The part- oligonucleotides fusions were annealed at the homologous 16 nt overhangs for 1 hour at 37°C, and used to transform electrocompetent *E*. *coli*.

pBAC0008 consisted of four parts: 1. the *E*. *coli/B*. *subtilis* shuttle vector backbone from pBAC0001; 2. the LacI repressor and isopropyl β-D-1-thiogalactopyranoside (IPTG) inducible P_grac_ promoter from pBAC0001, including a multiple cloning site; 3. the bidirectional strong *rrnB* T1 and T2 terminators; 4. the sgRNA expression module consisting of a kanamycin resistance gene flanked by AarI sites expressed under the control of the P_*veg*_ promoter, and the 'Cas9 handle' section of the sgRNA (5' truncated parts were prepared by PCR from the indicated template and oligonucleotides: 1. pBAC0001 with oMAP0010/0011; 2. pBAC0001 with oMAP0018/0019; 3. pING0001 with oMAP0024/0025; 4. pING0002 with oMAP0030/0031. Parts were ligated at 5’ and 3’ respectively with annealed oligonucleotides: 1. oMAP0008/0009 and oMAP0014/0015; 2. oMAP0016/0017 and oMAP0020/0021; 3. oMAP0022/0023 and oMAP0048/0049; 4. oMAP0050/0051 and oMAP0052/0053.)

pBAC0013 was constructed by converting the catalytically inactive *dcas9* gene from pdCas9-bacteria to active *cas9*. This was done by introducing mutations A10D, A840H and removing a BamHI site by PCR (oligonucleotides oMAP0062/0063/0064/0065/0066/0067) and a subsequent 3-part Gibson Assembly (pdCas9-bacteria was a gift from Stanley Qi (Addgene plasmid # 44249) [10].

Subsequently the catalytically active *cas9* gene was amplified with oligonucleotides oMAP0073/0074, introducing a BsaI site and XbaI recognition sites at the 5’ and 3’ end of the gene, respectively. The amplified *cas9* was digested with BsaI and XbaI, pBAC0008 was digested with BamHI and XbaI and both fragments were ligated with T4 DNA ligase, yielding pBAC0015.

To construct the subtilisin E overexpression plasmids pBAC0059, pBAC0060, pBAC0068 and pBAC0069, each subtilisin E variant and the wild type (WT) gene was amplified from chromosomal DNA derived from the relevant strain (Table 1) with oligonucleotides oMAP0186/0203, oMAP0200/0203, oMAP0186/0203, and oMAP0200/0203 respectively, each set containing 5’-BamHI and 3’-XmaI recognition sites. Oligonucleotides oMAP0203 also replaced the native GTG start codon with an ATG codon. The genes were cloned into pHT01 using the BamHI and XmaI recognition sites in the multiple cloning site of the plasmid.

### CRISPR-Cas9-Mediated gene editing in *B*. *subtilis*

#### Analysis of editing efficiency

For CRISPR-Cas9 mediated editing efficiency analysis, plasmids targeting the α-amylase *amyE* in *B*. *subtilis* were constructed. Plasmid pBAC0015 was digested with AarI (Thermo Fisher Scientific) to remove the kanamycin resistance gene and ligated to two annealed and phosphorylated DNA oligonucleotides of 24 nucleotides. The annealed oligonucleotides consisted of the 20 nucleotide protospacer region for targeting of the Cas9 protein and 4 nucleotides to generate a single stranded overhang for compatibility with the AarI digested vector. DNA phosphorylation was performed using T4 Polynucleotide Kinase according to the manufacturer’s instructions. The protospacer regions were identified using the online tool CRISPR-ERA: a comprehensive designer tool for CRISPR genome editing, (gene) repression, and activation, and selected based on the proximity to the desired modification [15]. The oligonucleotide pairs oMAP0089/0091, oMAP0125/0127 and oMAP0140/0142 yielded plasmids pBAC0027, pBAC0041 and pBAC0047 respectively. As a positive control for transformation efficiency, a sgRNA designed not to target the *B*. *subtilis* 168 chromosome was inserted into pBAC0015 with the oligonucleotide pair oMAP0145/0147, yielding pBAC0035. dDNA to introduce stop codons and repair the sgRNA-targeted Cas9 DSB were constructed by overlap extension PCR (OE-PCR) of two DNA fragments as described by Bryksin and Matsumura [16]. The desired edits were introduced in the homology overlap of the two DNA fragments. These included the silent mutation of the 5’-NGG-3’ protospacer adjacent motif (PAM) site to prevent continuous cutting by the RNA-guided Cas9 endonuclease. OE-PCR products from oligonucleotides sets oMAP0121/0122/0123/0124, oMAP0121/0122/0128/0129 and oMAP0121/0122/0143/0144 were used alongside pBAC0027, pBAC0041 and pBAC0047 respectively. Genome editing was carried out in triplicate by co-transforming naturally competent *B*. *subtilis* 168 with 200 ng CRISPR-Cas9/sgRNA plasmid DNA and 1 μg dDNA [17]. Transformants were spread on LB agar plates supplemented with 1% soluble potato starch (VWR) and chloramphenicol (5 μg/mL). IPTG was not added for *cas9* expression induction to limit cellular burden and it was found that basal expression under the P_grac_ promoter was sufficient for *cas9* expression. Effective knock-out of *amyE* by stop codon introduction was determined by staining transformation plates with iodine [18].

#### Editing of *aprE*

Using the approach described above, CRISPR-Cas9 mediated editing of the *aprE* gene in *B*. *subtilis* 168 was carried out. The oligonucleotide pairs oMAP0150/0151 and oMAP0156/0157 were ligated with pBAC0015 prepared as above, yielding plasmids pBAC0054 and pBAC0055 respectively. dDNA OE-PCR products from oligonucleotides sets oMAP0152/0153/0154/0155 and oMAP0158/0159/0160/0161 were used alongside pBAC0054 and pBAC0055 respectively. Editing was confirmed by PCR of the *aprE* gene with oligonucleotides oMAP0152/0161, followed by sequencing using oligonucleotides oMAP0158 and oMAP0155 for candidates edited with pMAP0054 and pMAP0055 respectively. The CRISPR-Cas9/sgRNA plasmid was removed from the edited strain by promoting plasmid loss in LB supplemented with 1 mM IPTG overnight. Here the presence of IPTG encourages curing by introducing the pressure of protein expression without the presence of chloramphenicol to retain the plasmid within the cell. Plasmid loss was confirmed by counter plating on LB agar plates with and without chloramphenicol before a subsequent round of editing. Following the second round of editing, these mutations and those introduced previously were confirmed to be present by sequencing (as above).

### Protein purification

The three subtilisin E variants and WT protein were overexpressed in the *aprE* knock-out strain BAC0114. BAC0114 was constructed by transformation of *B*. *subtilis* 168 with OE-PCR product containing a spectinomycin resistance cassette flanked by homology arms upstream and downstream of *aprE* (oligonucleotides oMAP0217/0218/0219/0220/0221/0222). Confirmation of *aprE* deletion was obtained by purification of the genomic DNA for BAC0114 and PCR of *ΔaprE*::*aad9* locus with oligonucleotides oMAP0670 (hybridising to *aad9*) and oMAP0671 (hybridising to the genome, upstream of the homology arm region). Additionally, PCR with oligonucleotide pair oMAP0217/0220 (hybridising to the extremities of the homology arm region) revealed the expected increase in product size for *ΔaprE*::*aad9* relative to the WT PCR product. This strain was then transformed with plasmids pBAC0059/0060/0068/0069, resulting in strains BAC0119/0120/0121/0122 respectively. These strains were grown for 24 hours at 37°C with agitation (250 rpm) in 20 mL LB supplemented with chloramphenicol. The supernatant was clarified and dialysed overnight into 100 mM Tris-HCl with 150 mM sodium chloride at pH 8. The dialysed supernatant was concentrated to 5 mL with Amicon Ultra-15 (10 kDa membrane (Merck)), filtered through a 0.2 μm filter (Sartorius). This was loaded onto a HiPrep Sephacryl S-200 HR, 120 mL 16/60 size exclusion column (GE Healthcare), eluted in the same buffer composition used for dialysis and fractions found to contain subtilisin E were pooled. Protein concentration was determined by absorbance at 280 nm.

### Thermal shift assay

The fluorescence-based thermal shift assay was used to determine the melting temperature (*T*_*m*_) of the subtilisin E variants and WT [19]. The assay reaction was prepared in a total volume of 20 μL (18 μL purified subtilisin E variant in purification buffer, 1 μL 100 mM calcium acetate and 1 μL 1:50 SYPRO Orange (Sigma-Aldrich)). The assay was performed with six technical replicates for each variant. The reaction was analysed using the Pikoreal 96 (Thermo Scientific) which recorded changes in fluorescence with increasing temperature (40°C—80°C in increments of 0.2°C, held for 6 seconds at each point).

### Subtilisin E activity assay

Subtilisin E variants and WT activity was determined by casein degradation as described by Cupp-Enyard [20].

### Residual enzyme activity tests

Residual enzyme activity was analysed by incubation of the purified enzymes at 55°C for 10, 20, 40, and 60 minutes prior to the activity assay.

## Results

### Analysis of CRISPR-Cas9 mediated gene editing efficiency

**Fig 1:**
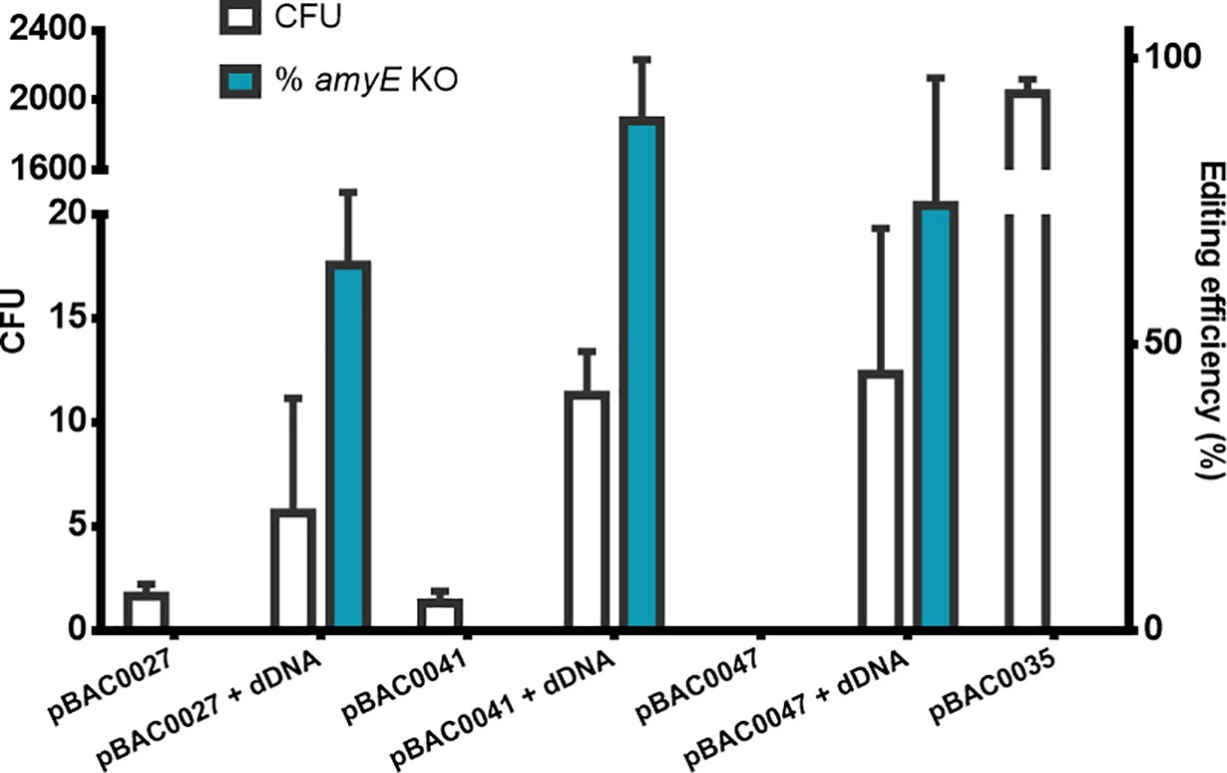
Number of transformants and editing efficiencies obtained following CRISPR-Cas9 genome editing of *amyE*. White bars represent the number of CFU obtained following transformation of three *amyE* targeting plasmids (pBAC0027/0041/0047) with or without editing template (dDNA) to introduce stop codons and repair the sgRNA-targeted Cas9 DSB. A plasmid containing a non-targeting sgRNA (pBAC0035) was transformed to monitor transformation efficiency. The blue bars represent the editing efficiency of the obtained CFU determined by observing the presence or absence of a halo following iodine staining of the starch-containing transformation plates. Error bars indicate the standard deviation between three transformations.

To analyse CRISPR-Cas9 mediated gene editing efficiency using our co-transformation system, the *amyE* gene encoding an α-amylase was selected for knock-out using a starch degradation assay. The three tested *amyE* targeting plasmids (pBAC0027/0041/0047) yielded on average an editing efficiency of 63.9%, 89.2% and 74.4% when co-transformed with dDNA (Fig 1).

### Rapid *in situ* modification of *aprE* with CRISPR-Cas9

**Fig 2:**
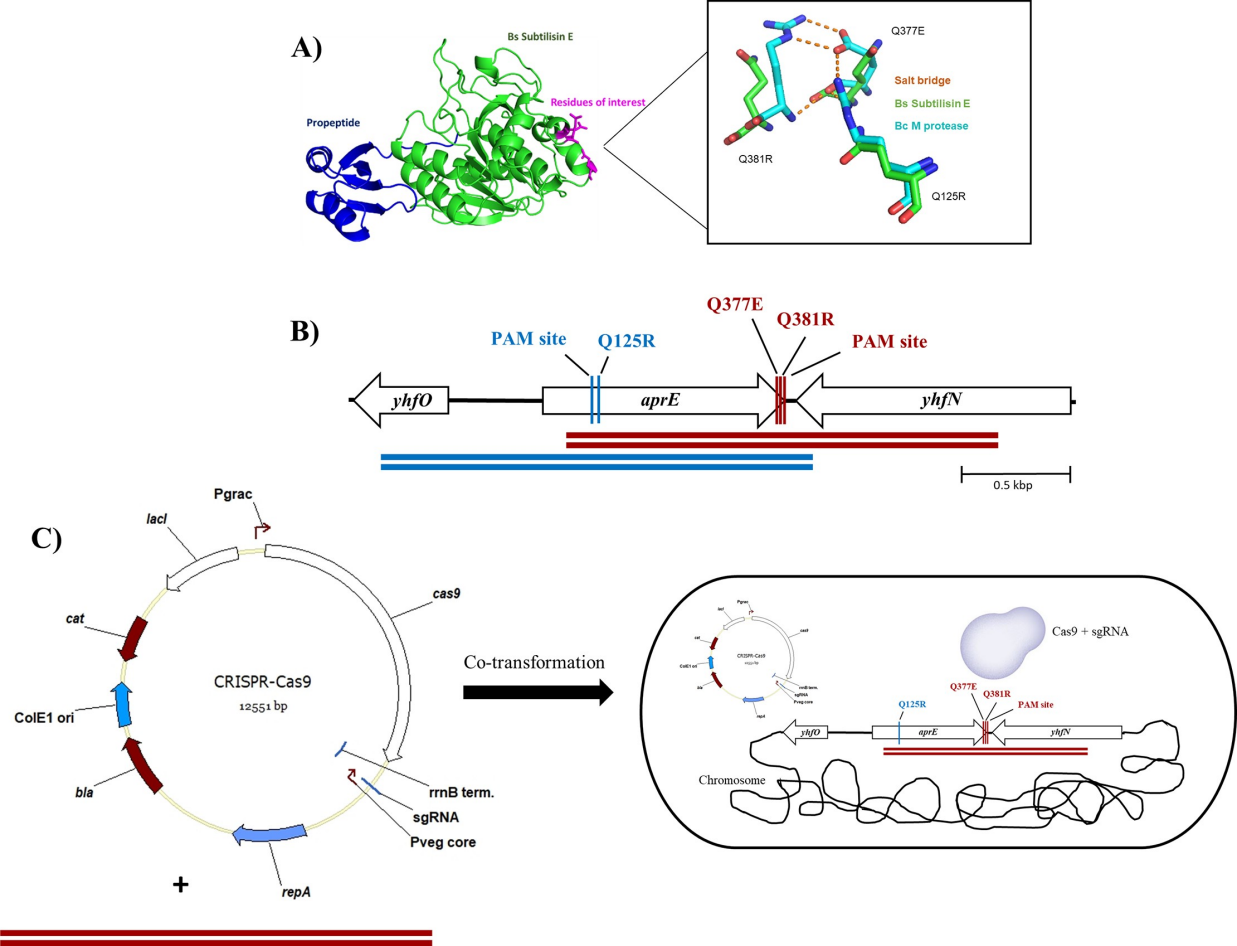
Design and CRISPR-Cas9 editing process of *aprE*. **A)** *B*. *subtilis* 168 subtilisin E crystal structure (green) and overlay with *B*. *clausii* M protease crystal structure (cyan), with the associated salt bridge (dashed yellow line). **B)** Design of the sgRNA and dDNA OE-PCR product for CRISPR-Cas9 genome editing. The two rounds of editing are described in blue and red, the PAM recognition sequences for each sgRNA was also targeted for disruption in each dDNA. **C)** Description of editing process where the second round of editing occurs following a blunt DSB by sgRNA guided Cas9.

The crystal structures for *B*. *subtilis* subtilisin E (PDB ID 1SCJ) and its *B*. *clausii* homolog, M-protease (PDB ID 1WSD), were overlaid using Swiss-Pdb viewer (Fig 2) [21–23]. The residues corresponding to the salt-bridge triad (R19-E271-R275) that have previously been shown to contribute towards M-protease thermotolerance were identified in the subtilisin E structure (Q125-Q377-Q381) [9].

A CRISPR-Cas9 mediated chromosome editing strategy was designed to introduce the mutations Q125R, Q377E and Q381R. pBAC0015 was altered to include the oligonucleotide pairs oMAP0150/oMAP0151 or oMAP0156/oMAP0157, yielding plasmids pBAC0054 (targeting Q125R locus) and pBAC0055 (targeting Q377E and Q381R locus) respectively. Due to their proximity, Q377E and Q381R modifications were combined into a single CRISPR-Cas9 mediated editing step with a single dDNA. Q125R was modified in a separate editing step. The desired edits, included the modification of the PAM site to prevent continuous cutting by the RNA-guided Cas9 endonuclease, were introduced in the homology overlap of the two DNA fragments prepared using PCR. These were subsequently combined using OE-PCR and co-transformed alongside the respective plasmid (Fig 2). OE-PCR products from oligonucleotides sets oMAP0152/0153/0154/0155 and oMAP0158/0159/0160/0161 were used alongside pBAC0054 and pBAC0055 respectively. Following an efficient curing process yielding strains BAC0094 (Q125R) and BAC0095 (Q377E and Q381R) respectively, a second round of editing using the second set of editing plasmid and dDNA yielded the final strain (BAC0097) containing all three modified residues. All screened colonies were found to contain the desired mutations following sequencing.

This system represents a rapid technique for *in situ* protein modifications within *B*. *subtilis* 168. Once the Cas9-sgRNA expression plasmid is prepared, the same region on the chromosome can be targeted with alternative, rapidly prepared, linear dDNA templates conveying novel modifications of interest.

### Thermotolerance of subtilisin E variants

**Fig 3:**
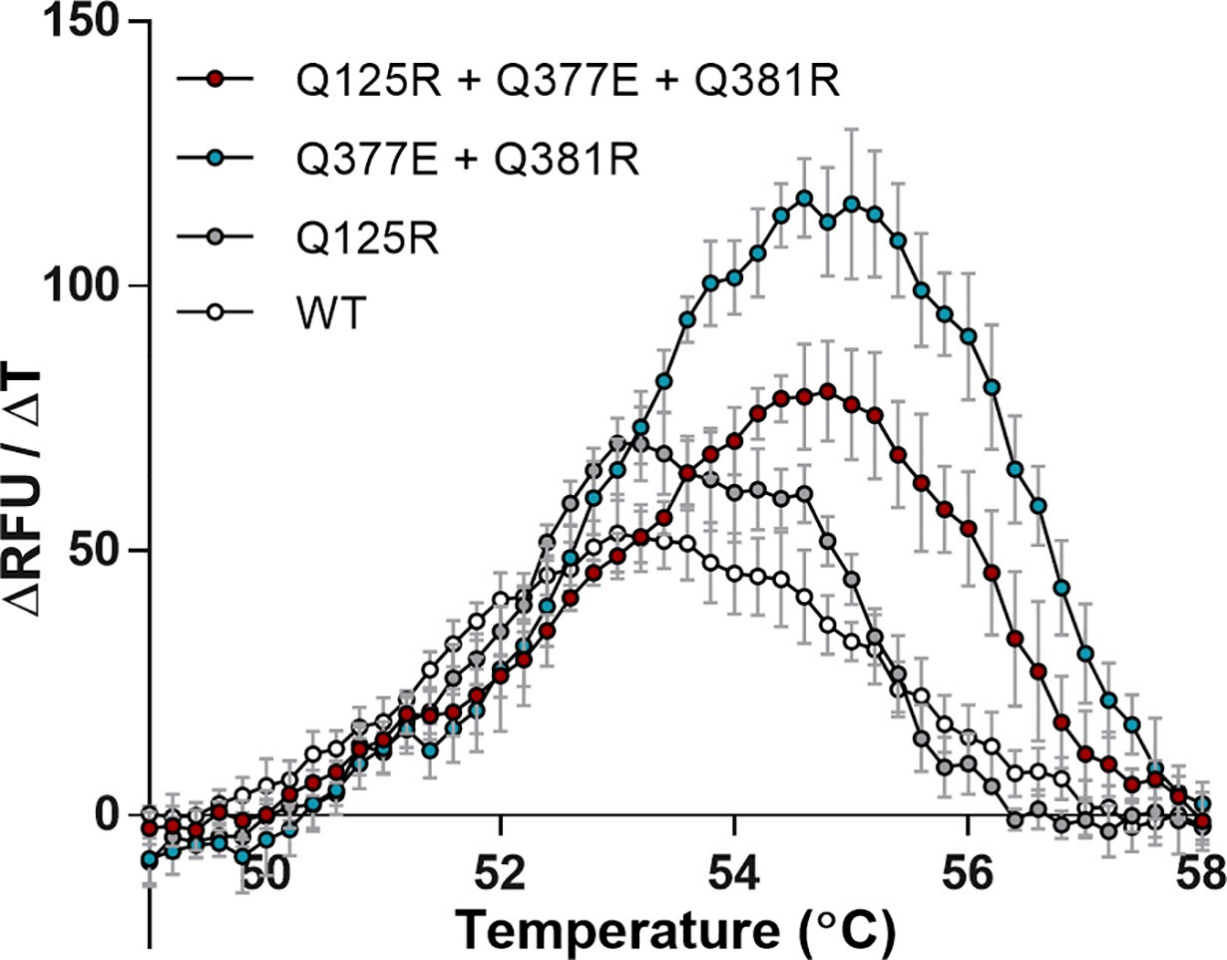
Thermal shift assay of the thermostability of each subtilisin E variant and WT. Increase in fluorescence was detected as hydrophobic regions of the protein were exposed as the protein denatured. The protein melting temperature of each variant and WT was calculated with a melt curve analysis, determining the peak rate of protein unfolding per temperature increase. Error bars indicate the standard deviation between six replicates.

The thermotolerance of purified subtilisin E variants and WT (purification described in materials and methods section) was analysed by the thermal shift assay (Fig 3). Variants Q377E+Q381R and Q125+Q377E+Q381R showed increased thermotolerance when compared to the WT and Q125R proteins, confirming the importance of residues E377 and R381 for protein stability at higher temperatures. In contrast, the Q125R variant showed no increase in thermotolerance, most likely as there is no mutated residue with which it can form salt bridge.

**Fig 4:**
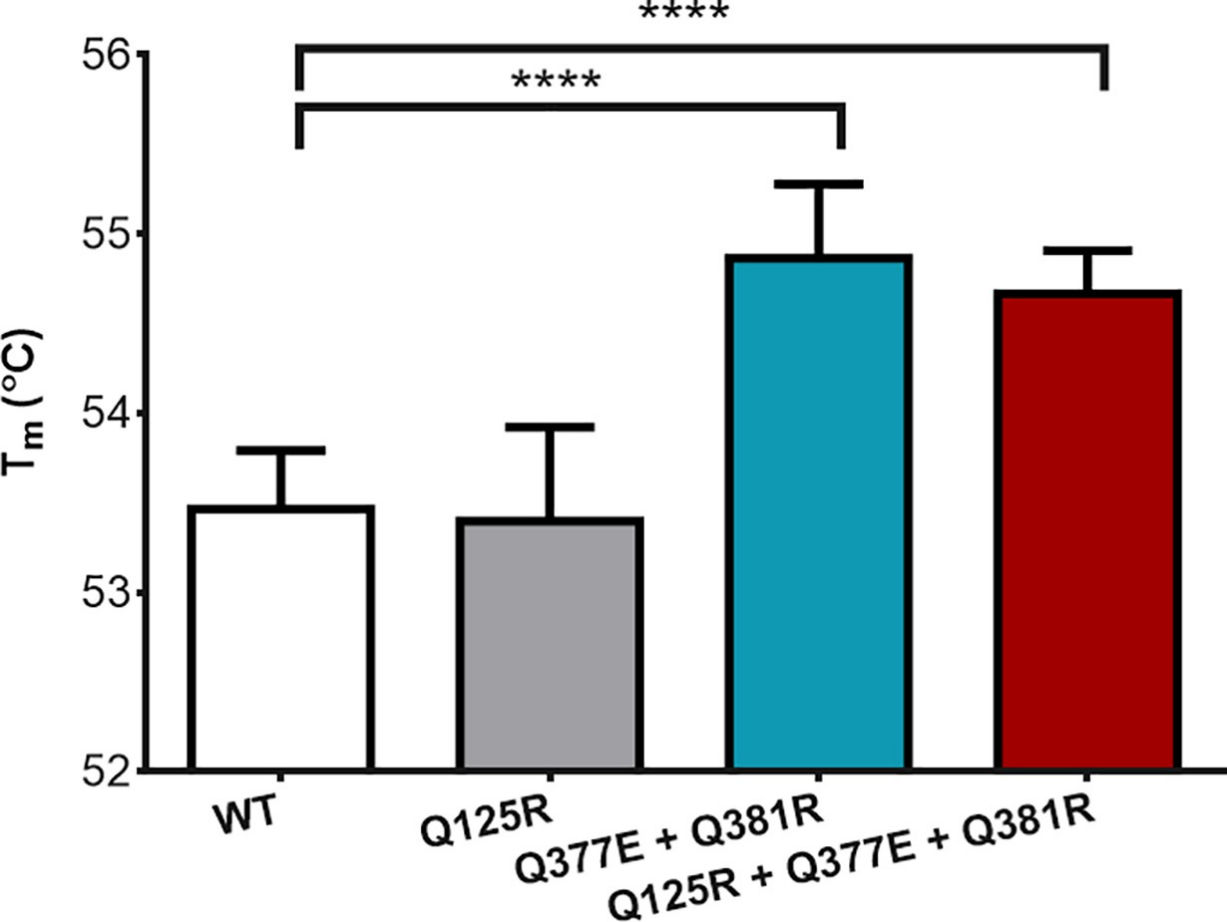
Average T_m_ between Subtilisin variants and WT replicates. The T_m_ of each subtilisin E variant and WT was calculated as the temperature at which the peak rate of protein unfolding was observed for each of six replicated. Error bars indicate the standard deviation. **** = p value summary (p = < 0.0001) following unpaired t test with Welch’s correction.

The average *T*_*m*_ for each variant was established by performing a melt curve analysis of the thermal shift assay data (Fig 3). An increase in *T*_*m*_ of up to 1.4°C was established and verified as statistically significant by use of an unpaired t test with Welch’s correction (Fig 4). The greatest increase in *T*_*m*_ of 1.4°C was found in the Q377E + Q381R variant (54.8°C, P value = < 0.0001), while the full salt bridge triad variant showed an increase of 1.2°C (54.6°C, P value = < 0.0001). The Q125R variant showed no significant difference in *T*_*m*_ when compared to the WT (53.4°C and 53.5°C respectively).

### Activity retention of subtilisin E variants

**Fig 5:**
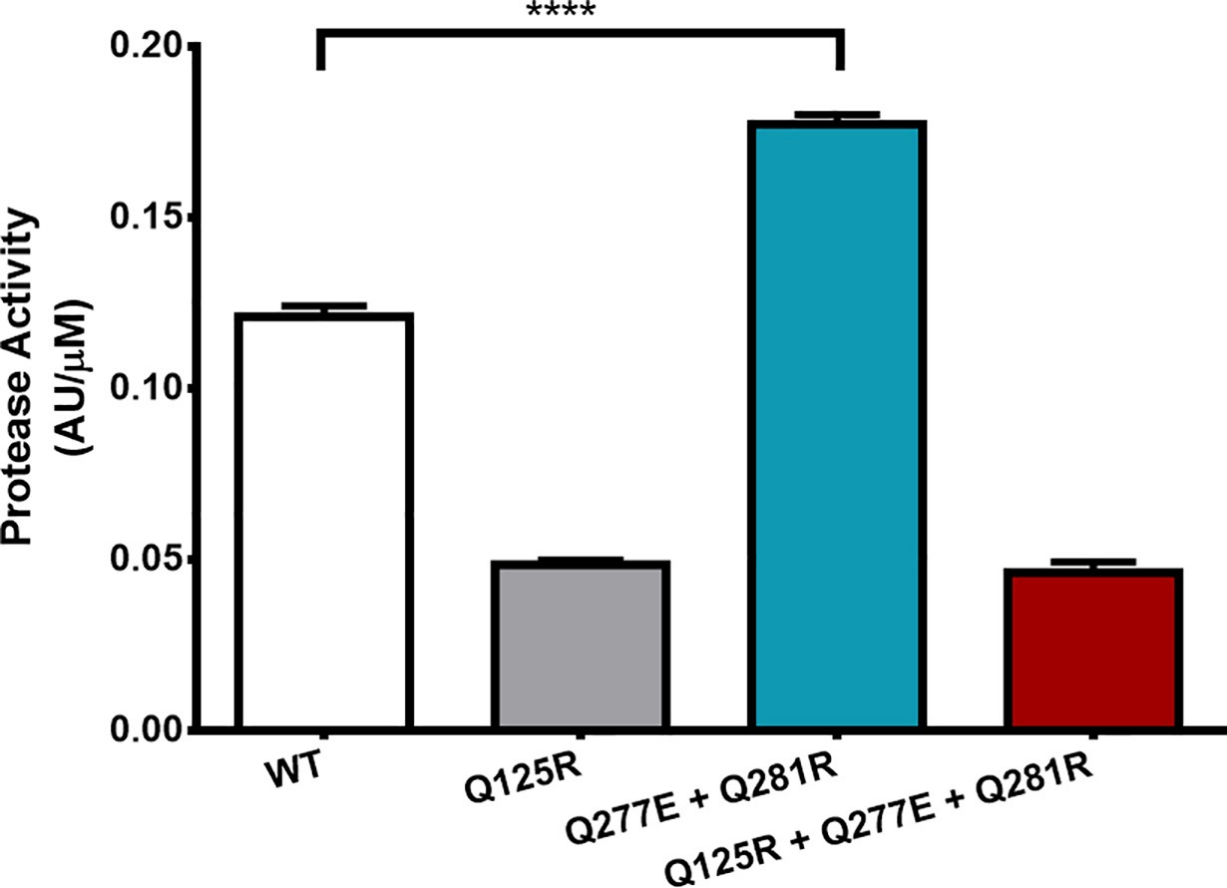
Protease activity assay. Protease activity at pH 7.5, 37°C for 10 minutes. Normalised for protein concentration determined by absorbance at 280 nm. Absorbance units (AU) determined at 660 nm. **** = p value summary (p = < 0.0001) following unpaired t test with Welch’s correction. Error bars indicate standard deviation between triplicates.

To ensure the introduced mutations had not negatively affected catalytic efficiency of the enzyme, the activity for each variant and the WT was established under neutral conditions (pH 7.5, 37°C) by measuring the degradation of casein using Folin’s reagent (Fig 5) [20]. Both the Q125R and Q125+Q377E+Q381R variants were 60% less active when compared to the WT, suggesting that the Q125R residue is important for catalytic activity. The Q377E+Q381R variant showed an increase in protease activity of 46.5% (P value = < 0.0001).

**Fig 6:**
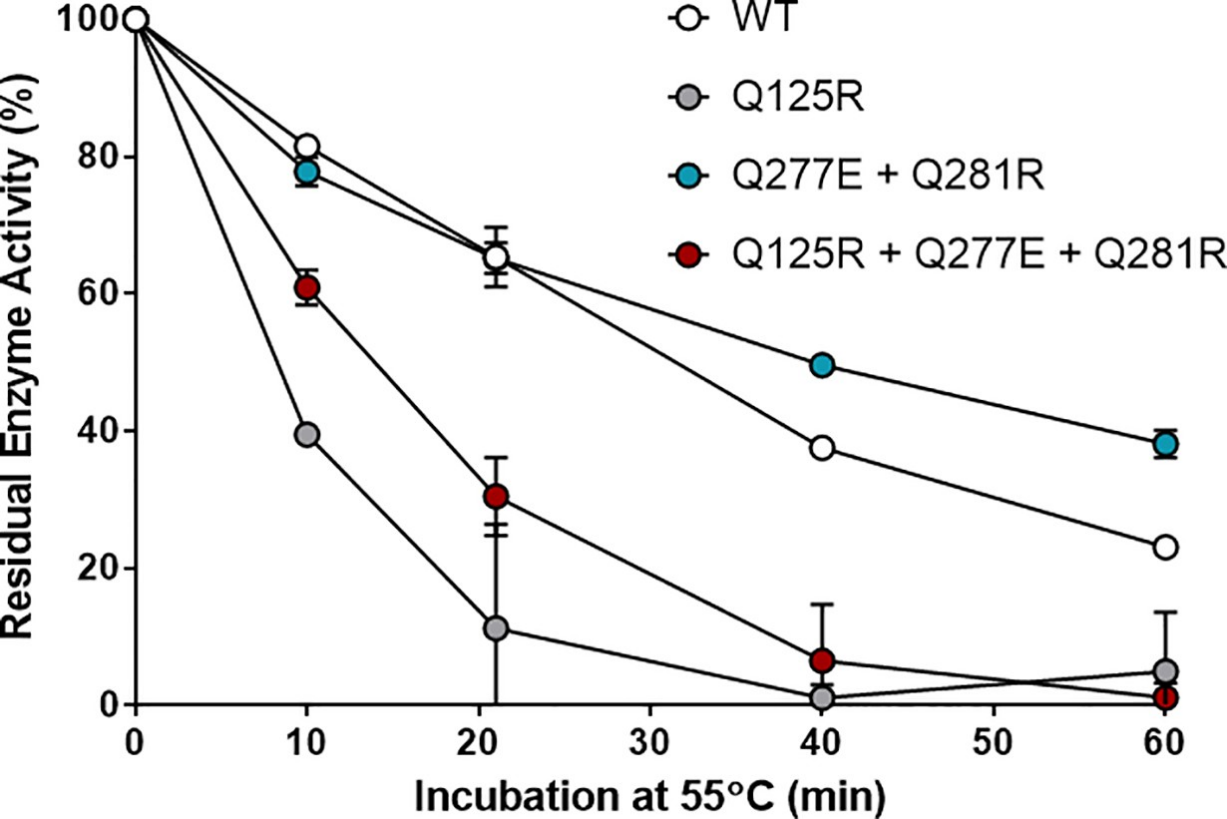
Residual protease activity assay. Residual protease activity under neutral conditions following incubation at 55°C for various lengths of time, determined by a casein degradation enzymatic assay. Error bars indicate standard deviation between technical triplicates.

Residual enzyme activity was measured following incubation of the enzyme variants at 55°C (Fig 6). Variant Q377E + Q381R was found to be the best at retaining its enzymatic activity when incubated for over 20 minutes. An improvement of 12.1% and 15.1% in activity relative to the WT following incubation for 40 and 60 minutes respectively was noted. The Q125 + Q377E + Q381R variant showed a 34.9% decreased in activity relative to the WT following 20 minutes incubation. Similarly, the Q125R variant showed a decreased in activity of 54.1% at 20 minutes incubation.

## Discussion

We have developed a *B*. *subtilis* specific, rapid, *in situ* protein engineering technique based on the ground breaking CRISPR-Cas9 technology. The rapid engineering of established and novel industrially relevant proteins yielding markerless, and potentially non-GMO compliant strains, is an attractive proposition for industrial biotechnology. This is due to the lowering in costs and time associated with product development and governmental regulatory compliance, while increasing the time a product is in production.

Initial exemplification of our system on the α-amylase encoding gene, *amyE*, showed an average editing efficiency of 76% across the three targeted PAM sites selected. While our co-transformation-based approach to genome editing may result in lower transformation efficiencies due to the requirement that both plasmid and dDNA molecules enter the cell, the rate of editing efficiency observed enables a high degree of confidence that most colonies obtained have been edited as designed. This system, where one round of plasmid construction is required and transformed alongside an easily prepared PCR product, represents an alternative to the *B*. *subtilis* CRISPR-Cas9 genome editing strategies published to date. These involve previous chromosome integration of the *cas9* gene and subsequent eviction of *cas9*/tracrRNA and crRNA expression cassettes after editing, or a two-step plasmid construction to introduce the sgRNA expression cassette and dDNA [2,3,5,6].

In under a week we carried out two rounds of *in situ* CRISPR-Cas9 editing of the subtilisin E gene, *aprE*. A salt bridge triad was introduced and the Q337E + Q381R variant showed an increase of 46.5% in subtilisin E activity, as well as a 1.4°C increase in thermostability. To the best of our knowledge these modifications have not been combined before in *B*. *subtilis* subtilisin E.

Interestingly, the Q125R + Q377E + Q381R variant did not retain its activity levels to the same level as the Q377E + Q381R variant. Indeed, in both variants where the Q125R mutation is introduced a drastic drop in enzyme activity retention was observed (Figs 5 and 6), indicating a vital catalytic activity role for residue Q125 within *B*. *subtilis* subtilisin E. A similar study of salt bridges in subtilisin E was performed by Erwin *et al*., where mutations Q125E + Q377E formed a salt bridge based on X-ray crystal structure, but resulted in a 1.2°C drop in thermotolerance [24].

In summary, this method for genomic modifications by CRISPR-Cas9 allows rapid, *in situ* protein engineering of industrially relevant strains. Furthermore, discovery and optimisation of new molecular biology tools such as the work presented here, increases the speed and efficiency at which novel biocatalysts can be developed for sustainable bioprocesses.

## Supporting information

S1 Fig Thermal shift assay of the thermostability of each subtilisin E variant and WT. Fluorescence increase observed as a result of protein unfolding and hydrophobic residue exposure during the thermal shift assay.
(TIF)

S1 File Thermo denaturation assay and melt curve analysis data. (XLSX)

S2 File Subtilisin E protein activity assay data. (XLSX)

S1 Table Oligonucleotides used in this study. (PDF)
