# Supplementary figures and images for: CRISPR-Cas9 In Situ engineering of subtilisin E in Bacillus subtilis
Source: PLoS One. 2019 Jan 7;14(1):e0210121. doi: 10.1371/journal.pone.0210121 (PMC6322745; doi:10.1371/journal.pone.0210121)

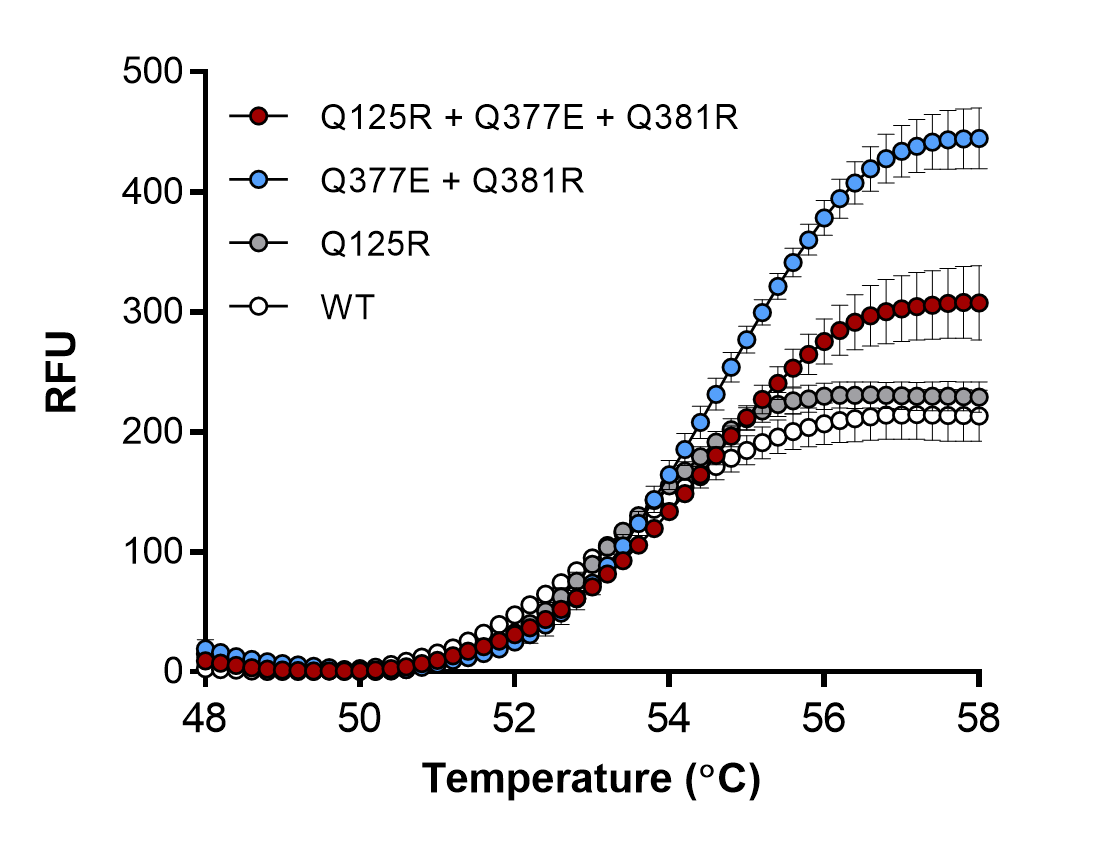

Supplement: S1 Fig — Fluorescence increase observed as a result of protein unfolding and hydrophobic residue exposure during the thermal shift assay. (TIF) [file pone.0210121.s001.tif]
