# Supplementary material for: CRISPR-Cas9 In Situ engineering of subtilisin E in Bacillus subtilis
Source: PLoS One. 2019 Jan 7;14(1):e0210121. doi: 10.1371/journal.pone.0210121 (PMC6322745; doi:10.1371/journal.pone.0210121)
Supplement: S1 Table — (PDF) [file pone.0210121.s004.pdf]

Table S1 - Oligonucleotides used in this study.

Oligonucleotide ID	DNA Sequence (5'-3')
oMAP0002	tattgggcgctctaccgcttcc
oMAP0003	gatatggacagaggagcaaa
oMAP0004	tttgctcctctgtccatatc
oMAP0005	aagcggtagagcgcccaatacgcat
oMAP0008	ctattcctaataagccgatattagcctcgtatgtt
oMAP0009	atcggcttattaggaa
oMAP0010	gaggaagcggccgcgctcttcgtagtccctgtactaataaaatcagg
oMAP0011	ttagatggatccgctcttcgggctggccgtcgttttacaac
oMAP0014	gccgtgaattcgagctcaggccttaactcacatt
oMAP0015	tgagctcgaattcac
oMAP0016	cagtgagcgcaacgcaattaatgtgagttaaggcc
oMAP0017	aattgcgttgcgctca
oMAP0018	gaggaagcggccgcgctcttcgctgcccgctttccag
oMAP0019	ttagataagcttgctcttcgggcggatccttcctcctttaattgg
oMAP0020	gccgactgctctagagtcgacgtccccggggcagc
oMAP0021	cgactctagagcagtc
oMAP0022	atctccatggacgcgtgacgtgaaaaaagcccgctcattaggcgggctgccc cggggacgt
oMAP0023	ccgcctaatgagcgggctttttttcacgtcacgcgtccatgga
oMAP0024	gaggaagcggccgcgctcttcagatgtttgtagaaacgcaaaaaggccatcc g
oMAP0025	ttagataagcttgctcttcgggcaacagaatttgcctggcg
oMAP0030	gaggaagcggccgcgctcttcgacggcaggtgttagaaaaactcatcgagc

oMAP0031	ttagataagcttgctcttcgggcagcaggtgtaaggaggcatcgtgaatgagccatattcaacgggaaacg
oMAP0048	gccaaaaaaagcaccgactcggtgccactttttcaag
oMAP0049	cgagtcggtgctttttt
oMAP0050	cgtagttttagagctagaaatagcaagttaaaataaggctagtccgttatcaacttgaaaaagtggcac
oMAP0051	ttgataacggactagccttattttaacttgctatttctagctctaaaact
oMAP0052	gccacatttattgtacaacacgagcccatttttgtcaacaacactggaacaaatggttgaaacatacgaggctaat
oMAP0053	tcaaccatttgttccagtgttgttgacaaaaatgggctcgtgttgtacaataaatgt
oMAP0062	tactcaataggcttagatatcggcacaaatagcgtcgg
oMAP0063	acgctatttgtgccgatatctaagcctattgag
oMAP0064	gtgattatgatgtcgatcacattgttccacaaagtttcc
oMAP0065	actttgtggaacaatgtgatcgacatcataatcac
oMAP0066	cgtaaaaaagactgggacccaaaaaaatatggtgg
oMAP0067	ccaccatattttttgggtcccagtctttttacg
oMAP0073	cactgaggtctctgatcctcatggataagaaatactcaataggc
oMAP0074	tcgtactctagattagtcacctcctagctgactcaaatcaatgc
oMAP0089	aaaccttttgatcgacggtgctgt
oMAP0091	atgtacagcaccgtcgatcaaaag
oMAP0121	atccggcgttctcatggcgg
oMAP0122	tattgaccgcagtgatagcc
oMAP0123	atgcatgaagaatggttcagcttttgatcgacggtgctgt
oMAP0124	acagcaccgtcgatcaaaagctgaaccattcttcatgcatgg
oMAP0125	aaacctttactgccgttattcgct
oMAP0127	atgtagcgaataacggcagtaaag
oMAP0128	aataacggcagtaaagttaagttttgaatcgttttgc

oMAP0129	aaacgattcaaaacttaactttactgccgttattcgctgg
oMAP0140	aaacgtcctgccagaaccaaatga
oMAP0142	atgttcatttggttctggcaggac
oMAP0143	gtttcagcactcgcagcctacggtcctgccagaaccaaatg
oMAP0144	catttggttctggcaggaccgtaggctgcgagtgctgaaac
oMAP0145	aaaccgagtcagctagctagcatg
oMAP0147	atgtcatgctagctagctgactcg
oMAP0150	aaacgcgctttaatttgagaaatg
oMAP0151	atgtcatttctcaaattaaagcgc
oMAP0152	aagaaaacgttggatagagc
oMAP0153	tcgagagtgaagagcaggcgctttaatttgagaaatgcc
oMAP0154	aaagcgcctgctcttcactctcgaggctacacaggctctaacg
oMAP0155	ttgacagagaacagagaagc
oMAP0156	aaacgcttctttttactattattg
oMAP0157	atgtcaataatagtaaaaagaagc
oMAP0158	ttaaacagacaatgagtgcc
oMAP0159	gcttctttttactattatcgtgcagctgcttctacgttgattaaccccttttcc
oMAP0160	cgataatagtaaaaagaagcagtttcctccatacctgcttc
oMAP0161	tttcgctgattacaacattgg
oMAP0186	gatcgtcccgggttattgtgcagctgcttgtacg
oMAP0200	gatcgtcccgggttatcgtgcagctgcttctacg
oMAP0203	gaggaaggatccgaatgagaagcaaaaaattgtgg
oMAP0217	tacaacattggtgacgctgcc
oMAP0218	gctaagcagaaggccatcctgtagtaaaaagaagcaggttcctccatacctgc
oMAP0219	cgttcaggcagtacgcctctttttctttaccctctcctttttaaaaaaaattcagag
oMAP0220	atcggcattttttttctgcctgc
oMAP0221	caggatggccttctgcttagc

oMAP0222	aaaagaggcgtactgcctgaacg
oMAP0670	caccatccttcggcaaatcc
oMAP0671	attgcgggaaatgcagtggc
